# Low-density lipoprotein cholesterol goal attainment in patients with clinical evidence of familial hypercholesterolemia and elevated Lp(a)

**DOI:** 10.1186/s12944-022-01708-9

**Published:** 2022-11-02

**Authors:** Andrea Schwarz, Ilja Demuth, Ulf Landmesser, Arash Haghikia, Maximilian König, Elisabeth Steinhagen-Thiessen

**Affiliations:** 1 Department of Endocrinology and Metabolic Diseases (Including Division of Lipid Metabolism), Charité – Universitätsmedizin Berlin, Corporate Member of Freie Universität Berlin and Humboldt-Universität zu Berlin, Biology of Aging Working Group, Augustenburger Platz 1, 13353 Berlin, Germany; 2 Department of Pediatrics, Charité -Universitätsmedizin Berlin, Division of Cardiology, Berlin, Germany; 3 BCRT – Berlin Institute of Health Center for Regenerative Therapies, Berlin, Germany; 4 Berlin Institute of Health at Charité-Universitätsmedizin Berlin, Berlin, Germany; 5 Department of Cardiology, Charité- Universitätsmedizin Berlin, Campus Benjamin Franklin, Berlin, Germany; 6 German Centre for Cardiovascular Research (DZHK), Partner Site Berlin, Berlin, Germany; 7 Institute of Clinical Chemistry and Laboratory Medicine, University of Rostock, Rostock, Germany

**Keywords:** LDL-cholesterol, Treatment goal achievement, Familial hypercholesterolemia, Lipoprotein (a), Coronary artery disease, Lipid-lowering treatment, Family history

## Abstract

**Background:**

Although potent lipid-lowering therapies are available, patients commonly fall short of recommended low-density lipoprotein cholesterol (LDL-C) levels. The aim of this study was to examine the relationship between familial hypercholesterolemia (FH) and elevated lipoprotein(a) [Lp(a)] and LDL-C goal attainment, as well as the prevalence and severity of coronary artery disease (CAD). Moreover, we characterized patients failing to meet recommended LDL-C goals.

**Methods:**

We performed a cross-sectional analysis in a cohort of patients undergoing cardiac catheterization. Clinical FH was determined by the Dutch Clinical Lipid Network Score, and Lp(a) ≥ 50 mg/dL (≈ 107 nmol/L) was considered elevated.

**Results:**

A total of 838 participants were included. Overall, the prevalence of CAD was 72%, and 62% received lipid-lowering treatment. The prevalence of clinical FH (probable and definite FH) was 4%, and 19% had elevated Lp(a) levels. With 35%, LDL-C goal attainment was generally poor. Among the participants with clinical FH, none reached their LDL-C target. Among patients with elevated Lp(a), LDL-C target achievement was only 28%. The prevalence and severity of CAD were higher in participants with clinical FH (86% prevalence) and elevated Lp(a) (80% prevalence).

**Conclusion:**

Most participants failed to meet their individual LDL-C goals according to the ESC 2016 and 2019 guidelines. In particular, high-risk patients with clinical FH or elevated Lp(a) rarely met their target for LDL-C. The identification of these patients and more intense treatment approaches are crucial for the improvement of CAD primary and secondary prevention.

**Supplementary Information:**

The online version contains supplementary material available at 10.1186/s12944-022-01708-9.

## Introduction

The burden of atherosclerotic cardiovascular disease (ASCVD) is still very high, and cardiovascular disease (CVD) is the leading cause of morbidity and mortality globally [1]. In Europe, more than 4 million deaths are due to CVD each year, accounting for approximately 45% of all deaths, according to the latest epidemiological update on CVD [2]. Coronary artery disease (CAD) alone accounts for 20% of all deaths [2].

Elevated low-density lipoprotein cholesterol (LDL-C) and other cholesterol-rich apolipoprotein B (apo B)-containing lipoproteins are key risk factors for ASCVD, and risk reduction is directly and positively correlated with the achieved absolute LDL-C reduction via lipid-lowering therapies [1, 3].

Steadily growing evidence has confirmed that lipoprotein(a) [Lp(a)] is likewise an independent risk factor for ASCVD. Hence, the updated European Society of Cardiology (ESC) and European Atherosclerosis Society (EAS) guideline for the management of dyslipidemias has included Lp(a) as a secondary treatment target in patients with otherwise optimally controlled risk factors [3, 4].

Lp(a) is a plasma lipoprotein that structurally resembles LDL-C but differs from LDL-C by containing an additional protein, apolipoprotein a [apo(a)]. Plasma levels of Lp(a) are mainly genetically determined by variations in the *APOA* gene and are only marginally influenced by diet, physical activity or other lifestyle habits, in contrast to other cholesterol-containing lipoproteins[4, 5]. Certain clinical conditions, such as familial hypercholesterolemia (FH) and chronic kidney disease, are associated with increased Lp(a) levels [6–8]. There is robust evidence showing that patients with higher plasma levels of Lp(a) are at greater risk of CAD and stroke [4, 7]. Currently, there are no approved pharmacologic therapies available that specifically target Lp(a) [9, 10].

Similar to elevated Lp(a), FH is independently associated with an increased risk of CVD. FH is caused by a monogenetic mutation and is characterized by defective clearance of LDL-C and premature coronary artery disease (CAD) [6]. FH can be diagnosed either by genetic testing for a causative mutation leading to elevated LDL-C levels or by using clinical criteria [6]. The Dutch Lipid Clinic Network (DLCN) created a scoring system based on clinical criteria assessing the probability of FH, ranging from unlikely, probable, possible to a definite diagnose of FH.

Although CAD mortality has declined and the management of dyslipidemia with lipid-lowering therapies (LLT) has improved over the last decades, mainly due to potent statin therapy [11], the guideline-recommended LDL-C reduction goals are vastly failed by most patients. Lipid goal attainment has been shown to be poor worldwide, including in Germany, ranging between 17 and 62% [12–14]. In particular, patients at high and very high cardiovascular risk regularly fail to reach the recommended lipid goals [14]. Unfortunately, those patients failing to meet their recommended goals remain at high risk of cardiovascular morbidity and mortality. Correct CVD risk evaluation is crucial for setting suitable lipid thresholds and preventing CVD and CVD progression. There are various scoring systems (the ESC-SCORE, the pooled cohort equations (PCE), or the PROCAM score, to name just a few) that include major CVD risk factors [3, 15, 16].

There is evidence that patients with genetic dyslipidemias such as FH are at greater risk of CVD and (recurrent) major adverse cardiovascular events (MACEs) than patients without a demonstrable genetic cause of dyslipidemia [18]. Arguably, at least 5% of all premature myocardial infarctions occur in FH individuals [19]. This can plausibly be explained by the long-standing exposure to elevated LDL-C and/or other cholesterol-rich apolipoprotein B (apoB)-containing lipoproteins since early childhood [18].

Reasons for the common failure in lipid goal attainment are not yet well described. A common argument is the lack of medication adherence due to side effects, especially to statins [13, 14, 17]. There is some evidence that high Lp(a) levels and FH impede the achievement of LDL-C goals [20, 21], but since Lp(a) status is often unknown, the association between Lp(a) and LDL-C goal attainment deserves further clarification.

The aim of this analysis was 1) to examine the current treatment situation of CAD patients with regard to LDL-C reduction in a large German tertiary care facility; 2) to determine the proportion of patients with diagnosed CAD failing to meet guideline recommended LDL-C targets; 3) to assess the association between dyslipidemias of (assumable) predominant genetic etiology (clinical FH and elevated Lp(a)) and LDL-C goal attainment; 4) to assess the predictive diagnostic value of family history regarding lipid disorders and CAD; and 5) to characterize patients with poor LDL-C control in a secondary prevention setting, to inform future studies/interventions aimed at improving lipid-lowering therapy and CAD prevention.

## Methods

### Study population and design

We performed a cross-sectional analysis of data from the LipidCardio study. The LipidCardio study is a prospective observational study examining patients who underwent elective coronary angiography for diagnostic or therapeutic reasons in the Department of Cardiology at Charité-Universitätsmedizin Berlin from October 2016 to March 2018. Patients who received catheterization for acute coronary syndromes were excluded. Demographic information, medical history, cardiovascular risk factors, extensive family history of first-degree relatives, medication plans before intervention, nonfasting lipid profiles, containing LDL-C (measured directly using an enzymatic colorimetric assay) high-density lipoprotein cholesterol (HDL-C), Lp(a) (using a turbidimetric assay by Roche Diagnostics), apo B, and HbA1c, medical examination results, including measurements of blood pressure (mmHg), pulse wave velocity (m/s), height (cm), weight (kg), and derived body-mass index (BMI, kg/m^2^), questionnaires on lifestyle habits (sports, smoking, alcohol) and coronary angiography results were collected. The rationale, design and methods of the LipidCardio study have been described in detail by König et al. [22]. Written informed consent was provided by all study participants, and the study was approved by the ethics committee at Charité-Universitätsmedizin Berlin (approval number: EA1/135/16).

**Fig. 1:**
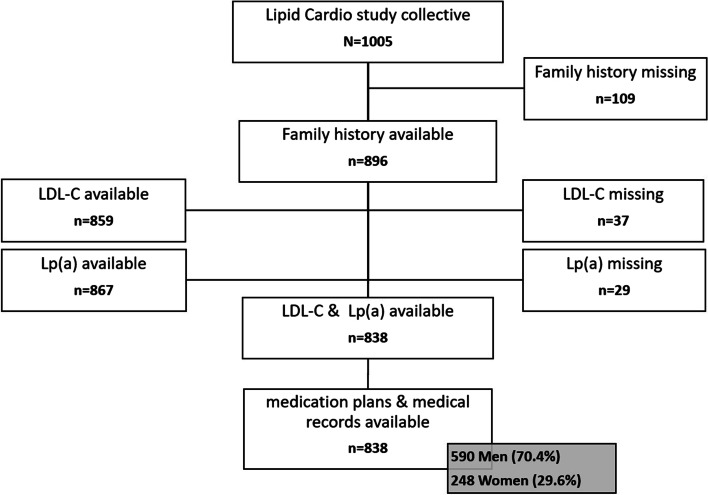
Flow chart of sample selection

In the present analysis, only participants for whom complete data containing family history, lipid profile, including LDL-C and Lp(a), medication, and diagnoses were available (Fig. 1) were included.

### Statin treatment intensity, projection of statin-naive LDL-C, and LDL-C target achievement

We inferred statin-naive LDL-C levels using published conversion factors (Supplementary table 1) [23–28]. Statin therapy was classified into low-intensity, moderate-intensity, and high-intensity groups based on expected LDL-C reductions of 20–32%, 36–50%, and 52–70%, respectively, depending on the statin type and dosage [23]. For 10-year cardiovascular risk estimation, we used the modified PROCAM score [29], and LDL-C target achievement was assessed based on the ESC/EAS guidelines for the management of dyslipidemias both from 2016, which were applicable at the study point, and from 2019 (currently valid). For very high-risk individuals, e.g., participants with CAD, the corresponding LDL-C target level was set at < 70 mg/dL; for high-risk individuals, the LDL-C target level was < 100 mg/dL; and for participants with intermediate or low risk, the LDL-C target level was < 115 mg/dL [30] according to the 2016 recommendations and < 55 mg/dL, < 70 mg/dL, and < 100 mg/dL with the recent recommendations [3].

### Definitions of dyslipidemia and CAD

Phenotypical familial hypercholesterolemia (FH) was ascertained according to the DLCN criteria. These were modified as described in the study of Langsted et al. because there was no information available on LDL-C levels in the offspring of participants, nor was there structured information on tendon xanthoma or corneal arcus in our participants and their relatives [31]. The probability of the FH diagnosis was operationalized into unlikely, possible, probable, or definite FH according to the modified DLCN criteria [31]. For the purpose of this analysis, participants with probable or definite FH according to the modified DLCN criteria were grouped together.

If Lp(a) levels were above 50 mg/dL (≈ 107 nmol/L), which equates to the fifth quintile in the examined study population, participants were considered to have “elevated” Lp(a) levels [4, 22].

CAD status was assessed by coronary angiography. Obstructive CAD was defined as a more than 50% obstruction in one of the major coronary arteries (left main coronary artery, left anterior descending artery [LAD], circumflex artery [RCX] and right coronary artery [RCA]). Obstructive CAD was categorized as 1-vessel, 2-vessel, or 3-vessel disease based on the number of coronary arteries with significant stenoses. Obstructions of the left main coronary artery were considered 2-vessel disease (LAD and RCX). Patients with coronary lesions or stenoses with less than 50% lumen obstruction were considered to have nonobstructive CAD, and “no CAD” was defined by normal coronary angiographic results.

### Statistical analyses

Participants with either probable or definite phenotypical FH and/or elevated Lp(a) were grouped together in some analyses. Proportions of LDL-C goal attainment were compared across phenotypical FH groups, between participants with and without elevated Lp(a) levels, and participants with and without genetic dyslipoproteinemia (probable and definite FH and/or elevated Lp(a)).

Statistical analyses were performed using the software package IBM SPSS 25.0, and figures were created using MS Excel 2013. Data are given in numbers (percentages), means and standard deviations (SDs), or medians and interquartile ranges (IQRs). We compared LDL-C goal attainment and the prevalence and severity of CAD between groups using the χ^2^-test for binary categorical variables, Kruskal‒Wallis test for categorical variables and the Jonckheere-Terpstra test for ordinal variables. Continuous variables were compared using t tests or analysis of variance (ANOVA) for more than two groups. Odds ratios (ORs) and 95% confidence intervals (CIs) were estimated using multivariable logistic regression. Kendall tau correlation was used to estimate associations between two ordinal variables. The statistical level of significance was set at *P* value < 0.05.

## Results

### Sample characteristics

Overall, 1005 participants were enrolled in the LipidCardio study between October 2016 and April 2018. Due to logistical reasons, complete family history, LDL-C, and Lp(a) levels were available from only 838 participants (Fig. 1.).

A total of 70.4% of participants were men (*n* = 590), and the mean (± SD) age was 69.4 (± 11.0) years. The majority (98.1%, *n* = 822) were of European ethnic origin. Overall, 71.5% (*n* = 599) had angiography-confirmed CAD. A total of 80.9% (*n* = 678) had a history of hypertension or were taking antihypertensive medication, 27.6% (*n* = 231) had been diagnosed with type 2 diabetes (T2D), 18.7% (*n* = 156) were current smokers, 61.6% (*n* = 516) were on lipid-lowering medication, and 25.9% (*n* = 216) were obese (BMI ≥ 30 kg/m^2^). The mean BMI was 27.9 (± 4.9) kg/m^2^ (Table 1).

**Table 1:** Baseline characteristics of the sample, total and according to familial hypercholesterolemia phenotype based on adjusted DLCN^a^ criteria

		**DLCN**^a^** criteria for phenotypical FH**			
	**total** ***N* = 838**	**unlikely FH** ***n* = 661**	**possible FH** ***n* = 140**	**probable FH** ***n* = 26**	**definite FH** ***n* = 11**
**Male sex**	590 (70.4)	469 (71)	96 (68.6)	18(69.2)	7 (63.6)
**Female sex**	248 (29.6)	192 (29)	44 (31.4)	8 (30.8)	4 (36.4)
**Age, years**	69 ± 11.0	71 ± 10.3	64 ± 11.8	59 ± 10.9	70 ± 10.2
**European ancestry**	822 (98.1)	650 (98.3)	135 (96.4)	26 (100)	11 (100)
**History of CAD**	431 (51.4)	332 (48.9)	85 (60.7)	16 (61.5)	7 (63.6)
**History of myocardial infarction**	262 (31.3)	185 (28.0)	62 (44.3)	10 (38.5)	5 (45.5)
**History of bypass surgery**	65 (7.8)	50 (7.6)	14 (10)	1 (3.8)	0 (0)
**Total obstructive CAD (new cases and previously established cases)**	599 (71.5)	458 (69.3)	109 (77.9)	21 (80.8)	11 (100)
**One vessel disease**	132 (15.8)	102 (15.4)	24 (17.1)	3 (11.5)	3 (27.3)
**Two vessel disease**	202 (24.1)	152 (23.0)	37 (26.4)	8 (30.8)	5 (45.5)
**Three vessel disease**	265 (31.6)	204 (30.9)	48 (34.3)	10 (38.5)	3 (27.3)
**Non-obstructive CAD**	104 (12.4)	88 (13.3)	14 (10)	2 (7.7)	0 (0)
**No apparent CAD**	135 (16.1)	115 (17.4)	17 (12.1)	3 (11.5)	0 (0)
**History of cerebrovascular event**	94 (11.2)	74 (11.2)	13 (9.3)	7 (26.9)	0 (0)
**Type 2 Diabetes**	231 (27.6)	187 (28.3)	34 (24.3)	6 (23.1)	4 (36.4)
**Hypertension**	678 (80.9)	539 (81.5)	109 (77.9)	21 (80.8)	9 (81.8)
**Current smoking**	156 (18.7)	95 (14.4)	44 (31.4)	12 (46.2)	5 (45.5)
**Pack Years**	30 ± 28.3	30 ± 28.9	30 ± 26.5	26 ± 17.4	34 ± 44.8
**Obesity**	216 (25.9)	160 (24.3)	47 (33.6)	7 (26.9)	2 (20)
**BMI (kg/m**^**2**^**)**	28 ± 4.9	28 ± 4.9	28 ± 5.2	28 ± 4.7	26 ± 4.2
**Systolic blood pressure (mmHg)**	135 ± 20.8	135 ± 21.0	133 ± 19.5	133 ± 22	150 ± 20.5
**Diastolic blood pressure (mmHg)**	78 ± 12.5	78 ± 12.9	78 ± 11.4	83 ± 13.3	85 ± 9.8

Values are mean ± SD and n (%), or median (25^th^-75^th^ percentile) unless stated otherwise

Of *n* = 838 observation values were missing in Obesity (*n* = 4), BMI (*n* = 4), Current smoking (*n* = 3), Pack Years (*n* = 3), Systolic blood pressure (*n* = 5), Diastolic blood pressure (*n* = 96)

^a^*DLCN* Dutch lipid clinic network, *FH *Familial hypercholesterolemia, *CAD* Coronary artery disease, *BMI *Body-mass index

#### Family history of CVD

A total of 30.4% of participants (*n* = 255) had a positive family history of premature CAD, i.e., a history of myocardial infarction, CAD, or cardiac death in first-degree relatives below the age of 55 years in men and 65 years in women. A total of 6.8% (*n* = 57) had a positive family history of stroke in first-degree relatives below the age of 55 years in men and 65 years in women. Overall, 34.5% (*n* = 289) had a positive family history of premature CVD.

#### Phenotypic familial hypercholesterolemia

According to the modified DLCN criteria, the majority of participants (78.9%, *n* = 661) were unlikely to have FH, 16.7% (*n* = 140) were possible cases of FH, 3.1% (*n* = 26) were probable FH cases, and only 1.3% (*n* = 11) were definite FH cases. Those with phenotypical FH (probable and definite FH) were on average significantly younger (62.2 vs. 69.8 years, *p* < 0.001) than participants without FH (unlikely and possible). The distribution of cardiovascular risk factors such as obesity, hypertension, and T2D was comparable between the groups. The proportion of current smokers was higher among participants with FH (45.9% vs. 17.4%, *p* < 0.001).

#### Lp(a)

A total of 19.3% of participants (*n* = 162) had elevated Lp(a) levels (≥ 107 nmol/L). There was no evidence of differences in sex, age, prevalence of hypertension, or current smoking among participants with and without elevated Lp(a) levels. The prevalence of T2D (21.0% vs. 29.1%, *p* < 0.027) and obesity (18.6% vs. 27.6%, *p* < 0.019) was significantly lower in patients with elevated Lp(a) than in participants without elevated Lp(a) (Supplementary Table 2a and b).

Overall, 21.5% of participants (*n* = 180) had either probable or definite phenotypic FH and/or elevated Lp(a) levels, i.e., evidence of a predominant genetic cause of dyslipidemia.

### Associations between a positive family history of CAD, Lp(a), FH, LDL-C levels, and CAD status

**Fig. 2:**
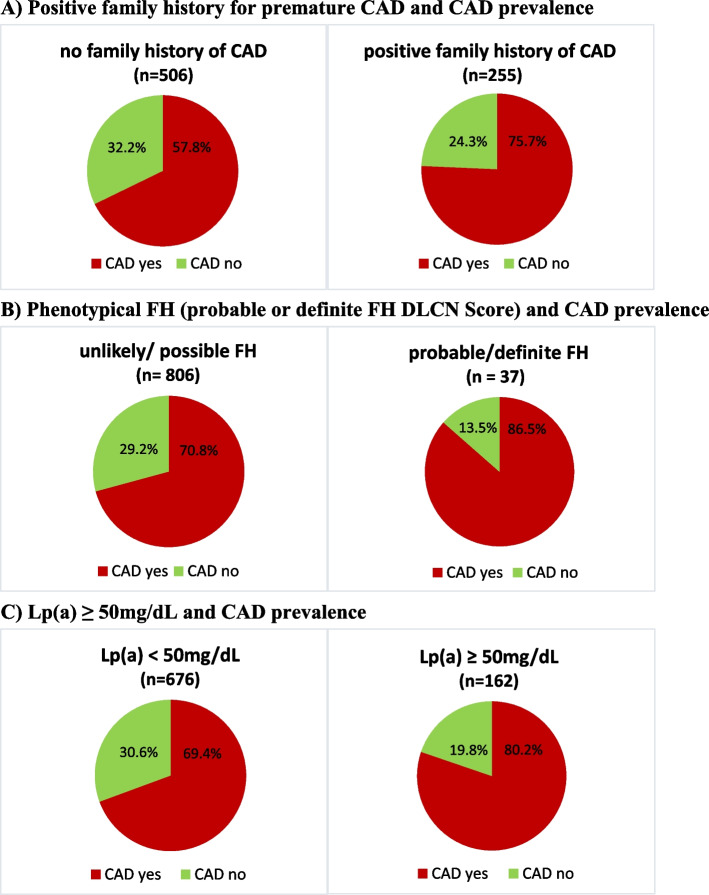
Prevalence of CAD according to. **A **presence of a positive family history of premature CAD in first degree relatives (men < 55 years, women < 65 years). **B** phenotypical (unlikely/possible vs. probable/definite) FH. **C **Lp(a) ≥ 50 mg/dL (107 nmol/L). Percentages of patients with CAD (red) and without CAD (green). *Patients not knowing family history were excluded (*n* = 77)

In participants with a positive family history of premature CAD, the prevalence of CAD was higher than in participants without a family history of CAD (75.7% vs. 67.8%, OR 1.5, CI 1.1 to 2.1, *p* = 0.024, Fig. 2A), and on average, CAD was significantly more severe with a family history of premature CAD (*p* for trend = 0.007) (Supplementary Fig. 1A). Remarkably, the mean LDL-C (98.0 mg/dL vs. 100.5 mg/dL), mean recalculated medication-naive LDL-C (143.3 mg/dL vs. 135.7 mg/dL), and median Lp(a) plasma concentrations (20.7 nmol/L vs. 17.9 nmol/L) were similar between patients with and without a positive family history of premature CAD. We did not find evidence of an association between a positive family history of premature CAD and elevated Lp(a) (*p* = 0.206).

The prevalence of CAD increased significantly with increasing FH score (*p* for trend = 0.005) (Table 1). In participants with phenotypical FH (probable or definite), the prevalence of CAD was significantly higher than in participants without phenotypical FH (87.5% vs. 70.8%, OR 2.6, CI 1.0 – 6.9, *p* = 0.039; Fig. 2B).

Likewise, CAD prevalence was significantly higher in participants with elevated Lp(a) levels than in participants with normal Lp(a) levels (80.2% vs. 69.4%, OR 1.8, CI 1.2 -2.7, *p* = 0.006; Fig. 2C). Median Lp(a) levels increased with increasing CAD severity (*p* for trend = 0.009, Table 2), and participants with elevated Lp(a) levels were more likely to be diagnosed with more severe CAD than participants with normal Lp(a) levels (*p* for trend = 0.002) (Supplementary Figure 1B).

**Table 2:** Mean LDL-C levels and median Lp(a) levels according to CAD severity

CAD severity	LDL-C (mg/dL)	Lp(a) (mg/dL)
	Mean (± SD)	Median (IQR)
**No CAD (*n* = 135)**	117.9 (± 36.7)	5.8 (< 2- 18)
**Non-obstructive CAD (*n* = 104)**	107.9 (± 40.3)	6.4 (< 2- 22.2)
**1-vessel CAD (*n* = 132)**	100.5 (± 42.1)	6.4 (< 2- 19)
**2-vessel CAD (*n* = 202)**	96.7 (± 40.0)	8.7 (< 2 – 40.9)
**3-vessel CAD (*n* = 265)**	87.7 (± 39.1)	11.1 (3.2 – 41.7)

Values are mean (± SD) for LDL-C and median (25^th^ to 75^th^ percentil) for Lp(a)

*CAD* status Coronary artery disease confirmed by coronary angiography

### Lipid profiles and lipid-lowering therapy

Mean levels for total cholesterol (TC), LDL-C, HDL-C, triglycerides (TG), and median levels of Lp(a) are shown in Table 3.

**Table 3:** Lipid profile and lipid-lowering therapy, total and according to familial hypercholesterolemia phenotype based on adjusted DLCN^a^ Criteria

		**DLCN**^a^** criteria score for phenotypical FH**			
	**total** ***N* = 838**	**unlikely FH** ***n* = 661**	**possible FH** ***n* = 140**	**probable FH** ***n* = 26**	**definite FH** ***n* = 11**
**Total cholesterol (mg/dL)**	169 ± 46.7	161 ± 41.6	184 ± 47.5	229 ± 60.9	252 ± 34.7
**LDL-C (mg/dL)**	99 ± 40.8	91 ± 34.6	120 ± 44.4	158 ± 48.3	179 ± 33.5
**Lp(a) (nmol/L)**	19 (5–72)	18 (5–70.7)	22 (8.3–61.2)	15 (9–141.4)	26 (5 -203.3)
**Lp(a) > 50 mg/dL (≈107 nmol/L)**	162 (19.3)	123 (18.6)	28 (20.0)	7 (26.9)	4 (36.4)
**HDL-C (mg/dL)**	50 ± 16.3	51 ± 16.3	50 ± 16.9	46 ± 10.6	49 ± 12.9
**Triglycerides (mg/dL)**	144 ± 86.5	133 ± 78.9	171 ± 99	212 ± 84.8	207 ± 123.0
**Lipid-lowering therapy (LLT)**	516 (61.6)	370 (56.0)	114 (81.4)	21 (80.8)	11 (100)

Values are mean ± SD and n (%), or median (25^th^-75^th^ percentile) unless stated otherwise

Of *n* = 838 observation values were missing in TC (*n* = 198), HDL-C (*n* = 2), TG (*n* = 224)

^a^*DLCN* Dutch Lipid Clinic Network, *FH* Familial hypercholesterolemia, *Lp*(a) Lipoprotein(a), *TC* Total cholesterol, *LDL-C* Low-density lipoprotein cholesterol, *HDL-C *High-density cholesterol, *TG *Triglycerides

There was evidence of sex differences for TC, LDL-C and HDL-C. Women had higher levels of TC (187.0 mg/dL vs. 161.6 mg/dL), LDL-C (112.4 mg/dL vs. 93.7 mg/dL), and HDL-C (59.1 mg/dL vs. 46.8 mg/dL) than men (*p* < 0.001). There was no evidence of a sex difference in the distribution of Lp(a) levels.

In the analyzed sample 516 (61.6%) participants received pharmacologic lipid-lowering therapy (LLT). Of these, 508 (98.4%) were treated with statins, 60 participants (11.6%) received ezetimibe, and only one participant used a proprotein convertase subtilisin/kexin type 9 (PCSK9) inhibitor (evolucumab). 465 participants (90.1%) were receiving a lipid-lowering monotherapy with statins, 8 participants (1.6%) were on a lipid-lowering monotherapy with only ezetimibe and 52 participants (10.1%) received a combination therapy of statin plus either ezetimibe or a PCSK9-inhibitor. A total of 322 participants (38.4%) did not receive any LLT. Participants with a preestablished diagnosis of CAD at study enrollment were more likely to be prescribed LLT (OR 8.9, CI 6.4 to 12.3, *p* < 0.001). However, 15.8% of participants (*n* = 68) with established CAD prior to study enrollment were not receiving LLT for secondary prevention.

Most participants were prescribed moderate-intensity statin treatment (66.5%, *n* = 343). A total of 9.5% (*n* = 49) received low-intensity statin treatment, and 24.0% (*n* = 124) received high-intensity LLT.

The higher the FH score, i.e., the probability of FH, the higher the LLT intensity was (*p* for trend < 0.001). No association was observed between elevated Lp(a) and LLT prescription and treatment intensity.

### LDL-C goal attainment

Overall, lipid goal achievement was very poor in the analyzed sample. Only 35.4% (*n* = 297) of the total study population met the recommended LDL-C levels according to the 2016 guidelines [30]. According to the more recent 2019 guidelines, only 17.4% of participants (*n* = 146) met their respective goals [3].

Considering only participants on previously established lipid-lowering medication, LDL-C goal attainment was 42.2% and 20.5% according to the 2016 and 2019 guidelines, respectively.

Overall, only 32.6% of those with diagnosed CAD (195 of 599 participants) reached their LDL-C targets. If those participants, whose first-time diagnosis of CAD coincided with study inclusion, and in whom LLT had only been established denovo (“new CAD”) were excluded, a slightly higher proportion (38.5%) of participants met their LDL-C goals (166 of 431).

We found evidence that with more intense LLT, more patients accomplished target levels (*p* for trend 0.016). However, only half of the participants (63 of 124) with high-intensity LLT reached their recommended LDL-C levels.

**Fig. 3:**
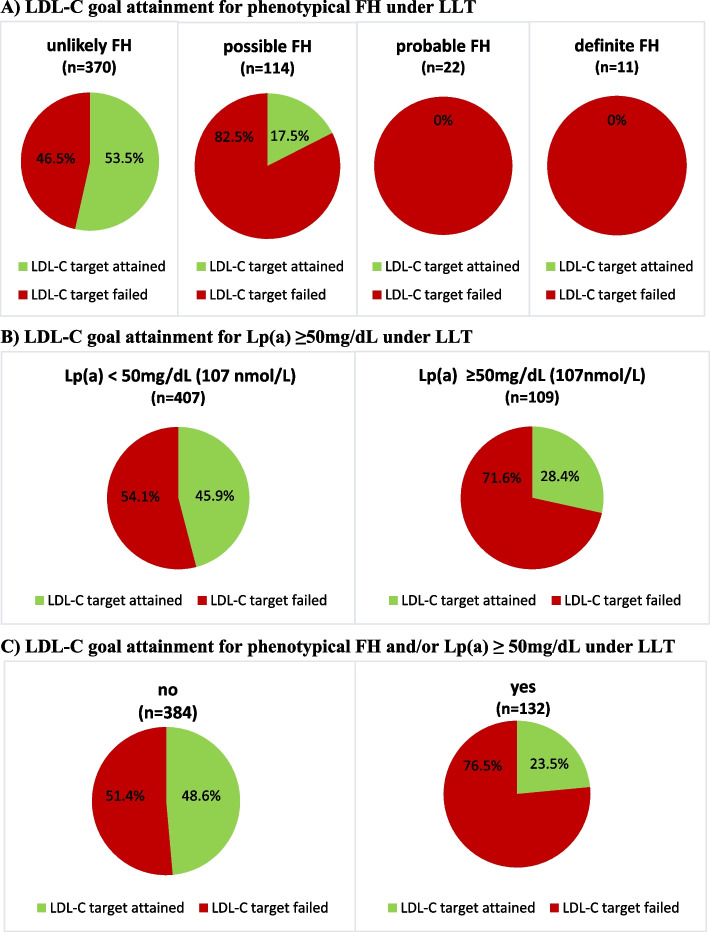
LDL-C target value attainment under current treatment. (**A**) probability of phenotypical FH by DLCN criteria (unlikely FH DLCN score < 3, possible FH DLCN score 3–5, probable FN DLCN score 6–8, definite FH DLCN score > 8). (**B**) Lp(a) ≥ 50 mg/dL (107 nmol/L). (**C**) prevalence of either probable or definite FH (DLCN criteria) and/or Lp(a) ≥ 50 mg/dL. Percentages of patients reaching (green) or failing (red) their individual LDL-C target based on ESC/EAS guidelines 2016 for risk groups (very high-risk LDL-C < 70 mg/dL, high risk LDL-C < 100 mg/dl, intermediate risk < 115 mg/dL)

With increasing FH score, the proportion of participants who reached target levels under LLT decreased (*p* for trend < 0.001) (Fig. 3A). Remarkably, in those with probable and definite FH (according to the DLCN score), no one met their recommended LDL-C levels.

Similarly, LDL-C target accomplishment was significantly less likely in participants with elevated Lp(a) levels than in those with nonelevated Lp(a) levels (OR 2.1 CI 1.4- 3.4, 28.4% vs. 45.9%, *p* < 0.001) (Fig. 3B). When we examined the relationship between Lp(a) plasma concentration quintiles and the proportion of LDL-C target attainment, there was evidence of an inverse linear trend (50% in the 1^st^ quintile, 54.8% in the 2^nd^ quintile, 38.5% in the 3^rd^ quintile, 41.8% in the 4^th^ quintile and 29.5% in the 5^th^ quintile (*p* for trend < 0.001)). The median distance to LDL-C target levels increased, with higher FH scores of 12.9 mg/dL, 45.8 mg/dL, 86.0 mg/dL and 109.5 mg/dL (participants grouped as unlikely, possible, probable, and definite FH, respectively) (*p* < 0.001). In an analogous manner, the median distance to the LDL-C target was higher in participants with elevated Lp(a) than in those with nonelevated Lp(a) (32.8 mg/dL vs. 19.3 mg/dL, *p* < 0.001). Consequently, when participants with either phenotypical FH (probable or definite FH score) or elevated Lp(a) were grouped together, target attainment was significantly less likely (48.6% vs. 23.5%, *p* < 0.001) compared to participants with neither clinical FH (unlikely or possible) nor elevated Lp(a) levels (Fig. 3C).

## Discussion

The present study reveals major treatment gaps in LLT in primary and secondary prevention of CAD. Notably, 16% of participants with previously known CAD were not receiving any LLT. Furthermore, overall LDL-C goal attainment rates were low, a finding that is consistent with other studies [13, 14]. Even among those with high-intensity lipid-lowering (statin) treatment, only half reached the targets set by the 2016 ESC/EAS guidelines for the management of dyslipidemias [32]. Application of the new target goals from the revised 2019 ESC/EAS guidelines resulted in even lower goal achievement rates [14, 33, 34].

While recommendations for LDL-C levels for optimal risk reduction tend to decrease further, current treatments fail to provide sufficient LDL-C lowering. A reason for treatment failure could be noncompliance [13, 14, 17]. Other reasons could be an underestimation of cardiovascular risk by the treating physicians and the reluctance to prescribe high-intensity treatment due to fear of potential side effects [35]. In fact, most participants on LLT received only moderate intensity treatment.

There are various tools to assess the risk of ASCVD in clinical practice, such as "PROCAM”, the “SCORE” by the ESC/EAS, and pooled cohort equations, to name just a few [3, 16, 29]. Most focus on TC and LDL-C as treatment targets. Other ASCVD biomarkers, such as Lp(a), non-HDL-C, and apo B, are considered secondary treatment targets in patients with otherwise optimally controlled risk factors but have not yet been established in risk estimation [3, 36]. There is growing evidence that non-HDL-C and apo B are especially better markers for risk estimation of CVD, as both are less prone to biased measurement than LDL-C [37–39]. To date, the determination of apo B and non-HDL-C as treatment targets in clinical trials is not common, and more investigation on this topic is needed. In our study sample, the use of combination therapy with statins and either ezetimibe or PCSK9 inhibitors was very scarce, which certainly contributed to the overall low LDL-C goal attainment, as statin monotherapy is known to be insufficient for LDL-C management in high- and very-high-risk patients, which was the case in our study cohort [34]. Since recruitment took place from 2016 to 2018, when PCSK9 inhibitors had been on the market for only 2 years, just one participant in our sample received a PCSK9 inhibitor. However, even today, the prescription of PCSK9 inhibitors is very limited, presumably due to high costs [17, 34]. Indeed, more widespread use of combination therapies, including the common use of PCSK9 inhibitors in (very) high-risk patients, could contribute to higher goal attainment rates [35]. It has been shown that adding a PCSK9 inhibitor to statin-based LLT can increase LDL-C goal achievement rates significantly, up to 68% [17, 35].

Secondary to the LDL-C-lowering effect, PCSK9 inhibitors, in contrast to statins, have been shown to reduce Lp(a) levels as well [10, 26], which also contributes to cardiovascular risk reduction. Unfortunately, this effect is mainly observed in patients with low Lp(a) levels, leaving patients with high CVD risk due to Lp(a) elevation unaffected [33].

Other Lp(a)-lowering therapies, such as an antisense oligonucleotide targeting apolipoprotein a (APO(a)-LRx), will be available soon. [20]. Furthermore, more innovative pharmacotherapeutic approaches are in progress [40]. Thus, the therapeutic armamentarium will be growing.

As another aim of the study, we examined the importance of probable or definite genetic causes of dyslipidemia, namely, phenotypical FH and elevated Lp(a), for achieving LDL-C targets. Both those with high Lp(a) and probable or definite FH were less likely to meet recommended lipid targets despite the use of intense lipid-lowering therapeutics by the majority. This finding is in line with studies, which have shown shortcomings in the effective treatment of FH patients [17, 35]. Insufficient LDL-C control in FH patients despite intense statin treatment might be explained by higher pretreatment LDL-C levels, which cannot be lowered to a sufficient level by statins alone [17]. As another explanation, mutations in the LDL-C receptor, which are responsible for the clinical FH phenotype, might also mitigate the effects of statins [41].

Regarding the poor LDL-C control in participants with elevated Lp(a), there are indications that when measuring the LDL-C concentration, the cholesterol contained in Lp(a) is also measured; thus, this part is quasi “statin-resistant”, i.e., it cannot be modified via statin therapy [31, 42]. To clarify this question, in-depth further studies need to be conducted.

It should be noted that in the clinical routine, LDL-C is often calculated based on TC, HDL-C, and TG, instead of being measured directly. Depending on the equation used, LDL-C determination is susceptible to bias, especially when TG levels are high [36]. For instance, when calculated by the Friedewald formula, LDL-C is underestimated if TG levels are high, which may result in undertreatment. Other equations, e.g., by Martin et al. or Sampson, have been shown to give more accurate estimates, as do direct measurements [43, 44].

We not only observed poor LDL-C management but also higher CAD prevalence and severity in participants both with phenotypical FH and elevated Lp(a). Arguably, life-long exposure to higher LDL-C levels in FH patients, mutation status and elevated Lp(a) levels, in addition to other established risk factors for ASCVD, are responsible for increased CAD incidence, prevalence, and progression in these patients [4, 18]. There is evidence that those with phenotypical FH or elevated Lp(a) are at particularly high risk of CAD and CAD progression [9]. The importance of early detection and decisive prevention cannot be overstated. Hence, guidelines suggest measuring Lp(a) levels at least once in life for risk stratification [3, 45].

The prevalence of phenotypical FH according to the DLCN criteria was higher in our sample than the assumed prevalence in the general population [6]. This may be explained by the selection of our sample. As patients with clinical FH are at greater risk of CAD, it seems reasonable that in a cohort of patients admitted for catheterization due to (suspected) CAD FH prevalence would be higher than in the general population. However, the distribution of Lp(a) and the proportion of elevated Lp(a) were in accordance with the reported distribution and proportion, respectively, in the total population [4].

The Frederickson dyslipidemia classification has long been the standard. However, novel approaches in the phenotypical classification of dyslipidemias are needed. Contemporary advances suggest a simplified classification based on the lipid perturbance found in routine lipid profiles and the inclusion of Lp(a) and apo B [46]. A recent study by Sampson et al. developed a new phenotypical classification system based on non-HDL-C and TG levels following the classical Fredrickson-like phenotypes. Depending on the classification, guidance for physicians on adequate treatment was derived [47]. However, this categorization also lacks the inclusion of Lp(a).

Another aim of this study was to assess the importance of a positive family history of premature CAD. Our findings confirm that the prevalence of CAD and, in particular, cases of more severe CAD were increased in patients with a positive family history of premature CAD. At the same time, patients with and without a positive family history had similar LDL-C levels and inferred treatment-naive LDL-C levels. In this regard, a previous study by Bachmann et al. showed that men with a family history of premature CAD had a 50% increased relative risk for CAD development than participants without a family history of CAD [48]. These findings underline the importance of obtaining a family history of premature CVD, as it may inform and guide the diagnostic procedure. In addition, taking a family history of premature CAD together with LDL-C measurement remains a simple way to identify patients with probable genetically caused dyslipidemias [49].

While there has been evidence for an association between Lp(a) levels and a family history of premature CVD from several studies in children and adolescents [50–52], we did not find a significant association between elevated Lp(a) and a positive family history of premature CVD in our sample and other studies in adults [53]. Reasons for this discrepancy remain unclear.

### Study strengths and limitations

A major strength of the study is the well characterized study population, including angiographic evidence of the presence of CAD, comprehensive lipid profiles and extensive family history assessment. A limitation is the lack of genetically verified FH diagnosis and the small proportion of phenotypical FH participants in the study sample. Another limitation is the absence of baseline LDL-C levels before the initiation of lipid-lowering treatment, so that treatment-naive had to be calculated instead, using published conversion factors. Furthermore, we had to use the PROCAM score for CVD risk estimation, as TC was not available in all cases. However, most of our cohort was already at very high risk given their CAD diagnosis, which made the risk calculation secondary in many cases. Last, there was no information about medication adherence or the duration of lipid-lowering treatment up to the time of the study.

## Conclusion

In conclusion, our results reflect the current unsatisfactory treatment situation in the management of dyslipidemias for (secondary) prevention of ASCVD, pointing out the discrepancy between clinical recommendations and reality. Goal attainment was even worse when there was evidence of a definite or probable genetic cause of dyslipidemia, i.e., phenotypical FH and elevated Lp(a). The identification of these high-risk patients from the heterogeneous group of CAD patients is crucial to provide suitable prevention strategies and appropriate therapy. For that, more innovative treatment approaches need to be established.

## Supplementary Information

**Additional file 1: Supplementary Figure 1.** severity of CAD for (A) patients with a positive family history of premature CAD in first degree relatives (men <55years, women < 65 years) *. (B) Lp(a) ≥ 50mg/dL (107nmol/L). Percentages of patients with no CAD, non-obstructive CAD, 1-vessel, 2-vessel, and 3-vessel CAD. *Participants not knowing family history were excluded (*n*=77).

**Additional file 2: Supplementary Table 1.** Calculated LDL cholesterol reduction by LLT.

**Additional file 3: Supplementary Table 2a.** Baseline characteristics of the sample, according to Lp(a) below and above upper quintile (50mg/dL ≈ 107 nmol/L). **Supplementary Table 2b. **Lipid profile of the sample, according to Lp(a)below and above upper quintile (50mg/dL ≈ 107 nmol/L).

## Data Availability

The datasets used and/or analyzed during the current study are available upon reasonable request (contact: ilja.demuth@charite.de).

## Abbreviations

Apo(a): Apolipoprotein A
ApoB: Apolipoprotein B
ASCVD: Atherosclerotic cardiovascular disease
CAD: Coronary artery disease
CVD: Cardiovascular disease
DLCN: Dutch Lipid Clinic Network
EAS: European Artherosclerosis Society
ESC: European Society of Cardiology
FH: Familial hypercholesterolemia
HDL-C: High-density lipoprotein cholesterol
LAD: Left anterior descending artery
LDL-C: Low-density lipoprotein cholesterol
Lp(a): Lipoprotein(a)
LLT: Lipid-lowering therapies
MACE: Major adverse cardiovascular events
PCSK9: Proprotein convertase subtilisin/kexin type 9
RCA: Right coronary artery
RCX: Circumflex artery
TC: Total cholesterol
TG: Triglycerides
T2D: Type 2 diabetes
