# Supplementary material for: Low-density lipoprotein cholesterol goal attainment in patients with clinical evidence of familial hypercholesterolemia and elevated Lp(a)
Source: Lipids Health Dis. 2022 Nov 2;21:114. doi: 10.1186/s12944-022-01708-9 (PMC9628073; doi:10.1186/s12944-022-01708-9)
Supplement: Supplementary file 1 — Additional file 1: Supplementary Figure 1. severity of CAD for (A) patients with a positive family history of premature CAD in first degree relatives (men <55years, women < 65 years) *. (B) Lp(a) ≥ 50mg/dL (107nmol/L). Percentages of patients with no CAD, non-obstructive CAD, 1-vessel, 2-vessel, and 3-vessel CAD. *Participants not knowing family history were excluded (n=77). [file 12944_2022_1708_MOESM1_ESM.docx]

**A) patients with a positive family history for premature CAD and CAD prevalence**

**B) Lp(a) > 50mg/dL and CAD severity**

**Supplementary Figure 1 severity of CAD** for

**(A)** patients with a positive family history of premature CAD in first degree relatives (men <55years, women < 65 years) *

**(B)** Lp(a) ≥ 50mg/dL (107nmol/L)

Percentages of patients with no CAD, non-obstructive CAD, 1-vessel, 2-vessel, and 3-vessel CAD

*Participants not knowing family history were excluded (n=77)
