# Supplementary material for: Low-density lipoprotein cholesterol goal attainment in patients with clinical evidence of familial hypercholesterolemia and elevated Lp(a)
Source: Lipids Health Dis. 2022 Nov 2;21:114. doi: 10.1186/s12944-022-01708-9 (PMC9628073; doi:10.1186/s12944-022-01708-9)
Supplement: Supplementary file 2 — Additional file 2: Supplementary Table 1. Calculated LDL cholesterol reduction by LLT. [file 12944_2022_1708_MOESM2_ESM.docx]

**Supplementary Table 1.** Calculated LDL cholesterol reduction by LLT

| **Drug dosage** | **calculated LDL-C reduction %** |
| --- | --- |
| **Fluvastatin 10mg** | 16.6% (28) |
| **Fluvastatin 20mg** | 23.1% (28,29) |
| **Fluvastatin 40mg** | 28.6% (28) |
| **Fluvastatin 80mg** | 33.3% (28,29) |
| **Simvastatin 10mg** | 28.6% (28) |
| **Simvastatin 20mg** | 33.3% (28,29) |
| **Simvastatin 30mg** | 35.5% |
| **Simvastatin 40mg** | 37.5% (28,29) |
| **Simvastatin 60mg** | 39.4% |
| **Simvastatin 80mg** | 41.2% (28,29) |
| **Simvastatin 100mg** | 42.9% |
| **Atorvastatin 10mg** | 37.5% (28) |
| **Atorvastatin 20mg** | 44.4% (24,28) |
| **Atorvastatin 30mg** | *47.4%* |
| **Atorvastatin 40mg** | 50% (24,28) |
| **Atorvastatin 60mg** | 52.4% |
| **Atorvastatin 80mg** | 54.5%(24,28,29) |
| **Rosuvastatin 5mg** | 42.9% (24,29) |
| **Rosuvastatin 10mg** | 44.4% (28) |
| **Rosuvastatin 20mg** | 47.4% (28,29) |
| **Rosuvastatin 40mg** | 52.4% (28,29) |
| **Pravastatin 10mg** | 23.1% (25,28) |
| **Pravastatin 20mg** | 23.1% (25,28) |
| **Pravastatin 40mg** | 28.6% (25,28) |
| **Ezetimibe 10mg** | 16.6% (26) |
| **Simvastatin 20mg + ezetimibe 10mg** | 50% (28) |
| **Simvastatin 40mg + ezetimibe 10mg** | 56.5% (24,28) |
| **Simvastatin 80mg + ezetimibe 10mg** | 58.3% (24,28) |
| **Atorvastatin 10mg + ezetimibe 10mg** | 52.8% (24) |
| **Atorvastatin 20mg + ezetimibe 10mg** | 53.9% (24) |
| **Atorvastatin 40mg+ ezetimibe 10mg** | 55.9% (24) |
| **Atorvastatin 80mg + ezetimibe 10mg** | 60.9% (24) |
| **Rosuvastatin 5mg + ezetimibe 10mg** | 59% (24) |
| **Rosuvastatin 10mg + ezetimibe 10mg** | 60.9% (24) |
| **Rosuvastatin 20mg + ezetimibe 10mg** | 66% (24) |
| **Rosuvastatin 40mg + ezetimibe 10mg** | 70% (24) |
| **Parvastatin 20mg + ezetimibe 10mg** | 37.9% (25) |
| **Fluvastatin 40mg + ezetimibe 10mg** | 41.2% |
| **Fluvastatin 80mg + ezetimibe 10mg** | 45.9% (26) |
| **Evolucumab** | 54.5% (27) |
| **Evolucumab +ezetimibe 10mg** | 70.6% (26,27) |

Values are calculated LDL-C reduction in % by each drug and dosage, literature references in parantheses
