# Supplementary material for: Low-density lipoprotein cholesterol goal attainment in patients with clinical evidence of familial hypercholesterolemia and elevated Lp(a)
Source: Lipids Health Dis. 2022 Nov 2;21:114. doi: 10.1186/s12944-022-01708-9 (PMC9628073; doi:10.1186/s12944-022-01708-9)
Supplement: Supplementary file 3 — Additional file 3: Supplementary Table 2a. Baseline characteristics of the sample, according to Lp(a) below and above upper quintile (50mg/dL ≈ 107 nmol/L). Supplementary Table 2b. Lipid profile of the sample, according to Lp(a)below and above upper quintile (50mg/dL ≈ 107 nmol/L). [file 12944_2022_1708_MOESM3_ESM.docx]

**Supplementary Table 2a.** Baseline characteristics of the sample, according to Lp(a) below and above upper quintile (50mg/dL ≈ 107 nmol/L)

|  | Lp(a) < 50mg/dL  (107 nmol/L) | Lp(a) ≥ 50mg/dL  (107 nmol/L) |
| --- | --- | --- |
|  | **n = 676** | **n = 162** |
| Men | 483 (71.4) | 107 (66.0) |
| Women | 193 (28.6) | 55 (34.0) |
| Age, years | 69±11.2 | 70±10.0 |
| History of CAD | 333 (49.3) | 98 (60.5) |
| History of myocardial infarction | 201 (29.7) | 61 (37.7) |
| History of bypass surgery | 43 (6.4) | 22 (13.6) |
| Total obstructive CAD (new cases and previously established cases) | 469 (69.4) | 130 (80.2) |
| One vessel disease | 112 (16.6) | 20 (12.3) |
| Two vessel disease | 154 (22.8) | 48 (29.6) |
| Three vessel disease | 203 (30.0) | 62 (38.3) |
| Non-obstructive CAD | 90 (13.3) | 14 (8.6) |
| No apparent CAD | 117 (17.3) | 18 (11.1) |
| History of cerebrovascular event | 70 (10.4) | 24 (14.8) |
| Diabetes mellitus | 197 (29.1) | 34 (21.0) |
| Hypertension | 548 (81.1) | 130 (80.2) |
| Current smoking | 126 (18.7) | 30 (18.5) |
| Pack Years | 30±28.5 | 29±27.7 |
| Obestity | 186 (27.6) | 30 (18.6) |
| BMI (kg/m²) | 28±5.0 | 27±4.5 |
| Systolic blood pressure (mmHg) | 135±10.8 | 134±21.1 |
| Diastolic blood pressure (mmHg) | 78±12.0 | 79±14.3 |

Values are mean ±SD and n (%) unless stated otherwise. Of n=838 observation values were missing in Obesity (n=4), BMI (n=4), Current smoking (n=3), Pack Years (n=3), Systolic blood pressure (n=5), Diastolic blood pressure (n=96).

CAD, coronary artery disease; BMI, body-mass index.

**Supplementary Table 2b.** Lipid profile of the sample, according to Lp(a) below and above upper quintile (50mg/dL ≈ 107 nmol/L)

|  | Lp(a) < 50mg/dL  (107 nmol/L) | Lp(a) ≥ 50mg/dL  (107 nmol/L) |
| --- | --- | --- |
|  | **n = 676** | **n = 162** |
| TC (mg/dL) | 166±46.7 | 179±45.6 |
| LDL-C (mg/dL) | 97±30.2 | 107±42.6 |
| HDL-C (mg/dL) | 50±16.0 | 53±17.2 |
| TG (mg/dL) | 147±90.4 | 135±67.9 |
| Lipid-lowering therapy | 407 (60.2) | 109 (67.3) |

Values are mean ±SD and n (%) unless stated otherwise. Of n=838 observation values were missing in TC (n=198), HDL-C (n=2), TG (n=224).

Lp(a), lipoprotein(a); TC, total cholesterol; LDL-C low-density lipoprotein cholesterol; HDL-C, high-density cholesterol; TG; triglycerides.
